# Defect Engineering Strategies Toward Controlled Functionalization of Solution‐Processed Transition Metal Dichalcogenides

**DOI:** 10.1002/smsc.202100122

**Published:** 2022-02-16

**Authors:** Stefano Ippolito, Paolo Samorì

**Affiliations:** ^1^ CNRS ISIS UMR 7006 University of Strasbourg 8 Allée Gaspard Monge 67000 Strasbourg France

**Keywords:** defect engineering, functionalization strategies, hybrid functional materials, solution processing, transition metal dichalcogenides

## Abstract

Solution‐processed transition metal dichalcogenides (TMDs) are attracting unceasing attention owing to their wide‐ranging portfolio of physicochemical properties, making them prime candidates for low‐cost and real‐life applications in (opto)electronics, (bio)sensing, and energy‐related technologies. The performance of TMD‐based devices is strictly interconnected with the inherent features and quality of the materials, which should be tuned in view of their ultimate application. In this regard, the device performance is hitherto undermined by the presence of structural defects inherited from both the bulk systems and the exfoliation procedures. To overcome this limitation, a notable research effort has been devoted to the development of molecular strategies taking advantage of the defective nature of solution‐processed TMDs, in order to meticulously tailor their physicochemical properties and expand the range of applicability. In this perspective, some of the most enlightening advances regarding the functionalization approaches exploiting TMD structural defects are presented, introducing the typical “imperfections” encountered in 2D crystal lattices (with different dimensionality, ranging from 0D to 2D) as well as discussing their in situ/ex situ generation methods. Finally, we highlight the future directions, challenges, and opportunities of defect engineering in TMDs by offering guidelines to boost the progress of 2D materials science and related technology.

## Graphical abstract

Structural defects in solution‐processed transition metal dichalcogenides (TMDs) are typically related to inferior material properties and device performance. This perspective provides a timely guideline on defect engineering in TMDs to control the fabrication of hybrid multifunctional materials with enhanced functionalities, thereby paving the way toward new disruptive technologies.

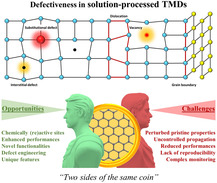

## 1 Introduction

The discovery of the outstanding physical properties of 2D materials (2DMs)^[^ ^1^, ^2^ ^]^ represented a veritable breakthrough in materials‐ and nano‐science, paving the way to the development of unprecedented technologies.^[^ ^3^ ^]^ The observation of exotic quantum effects and phenomena in the atomically thin limit has promoted the ever‐growing diffusion of 2DMs in applications spanning from (opto)electronics^[^ ^4^ ^]^ and (photo)catalysis^[^ ^5^ ^]^ to (bio)sensing and biomedicine.^[^ ^6^ ^]^ Graphene, the ancestor of the 2DM family, exhibits superlative physical and chemical properties,^[^ ^7^, ^8^ ^]^ attracting enormous interest from both academia and industry. However, the lack of bandgap imposes severe restrictions on its switching ability in devices such as (photo)transistors and diodes,^[^ ^2^ ^]^ hindering the massive diffusion of graphene in (opto)electronics. In this regard, semiconducting transition metal dichalcogenides (TMDs) have become the flagship alternative to overcome such a bottleneck, offering tunable bandgaps in the whole visible spectrum (approximately from 1 to 3 eV)^[^ ^9^ ^]^ owing to their thickness‐dependent electronic characteristics.^[^ ^10^, ^11^ ^]^ During the last decade, fast‐moving research has been carried out to develop novel methods and techniques for the large‐scale production of TMDs, characterized by diverse cost, scalability, and yield depending on the chosen operating conditions.^[^ ^12^, ^13^ ^]^ Solution processing represents a valuable protocol to attain high‐concentration and high‐volume TMD dispersions, commonly referred to as “inks,” where bulk crystals are exfoliated and dispersed in a specific solvent *via* energy transfer that overcomes the weak van der Waals (vdW) interactions holding together adjacent sheets within multilayered structures.^[^ ^14^, ^15^, ^16^ ^]^ The high throughput achieved by solution processing promotes the wide use of TMDs in many different applications, exploiting pristine or hybrid materials in the form of dispersions, coatings, and thin‐films produced by various deposition techniques, including inkjet printing, spray coating, roll‐to‐roll, spin‐coating, and drop‐casting.^[^ ^17^ ^]^ Although the production methods already contribute to affect the quality and characteristics of solution‐processed TMDs (i.e., purity, aspect ratio), many research endeavors have also been devoted to the development of molecular strategies to meticulously tailor on demand their properties.^[^ ^18^, ^19^ ^]^ The physisorption and chemisorption of molecules and assemblies thereof onto 2D crystals are driven by a variety of mechanisms, whose versatility offers a powerful toolbox in such an exciting area of 2DM science.^[^ ^20^ ^]^

As a result of the interplay between thermodynamics and kinetics of their processing, all materials possess structural defects that significantly affect their properties. Defects residing in 2D crystals can be classified according to their dimensionality,^[^ ^21^ ^]^ such as 0D (point defects, dopants), 1D (grain boundaries, edges, in‐plane heterojunctions), and 2D (stacking of vdW solids, wrinkling, folding, scrolling).

However, supported by thermodynamic considerations, 0D defects are the most abundant stoichiometric deficiencies in TMDs, especially chalcogen vacancies that are mainly located at the flake edges and whose formation energy amounts to a few eV (≈2 eV in the case of sulfur vacancies, *V*_S_).^[^ ^21^ ^]^ It is worth mentioning that defects in 2DMs can be either unintentionally formed during production steps (in situ) or deliberately introduced by processing (ex situ), thereby altering their final characteristics.^[^ ^18^, ^22^ ^]^ In this regard, they can be considered the archetype of “Janus Bifrons” in materials science: defects drastically affect, and in many cases deteriorate, the pristine electrical, mechanical, photonic, and thermal properties of TMDs, as thoroughly discussed in previous reviews^[^ ^23^, ^24^ ^]^; yet, at the same time, they can endow TMDs with singular features that are absent in perfect crystals. Above all, defects offer unique opportunities for functionalization strategies by molecular chemistry approaches, by acting as primary (re)active sites in 2DMs.^[^ ^19^, ^20^ ^]^

In this perspective, we review and highlight the recent seminal works on defect generation in TMDs, as well as the most enlightening and promising functionalization strategies taking advantage of their inherent defectiveness. Finally, we discuss the challenges and opportunities of defect engineering to produce hybrid multifunctional materials based on solution‐processed TMDs, providing general guidelines to advance the knowledge toward novel technological breakthroughs.

## 2 Solution‐Processed TMDs: Production Methods

**Figure 1:**
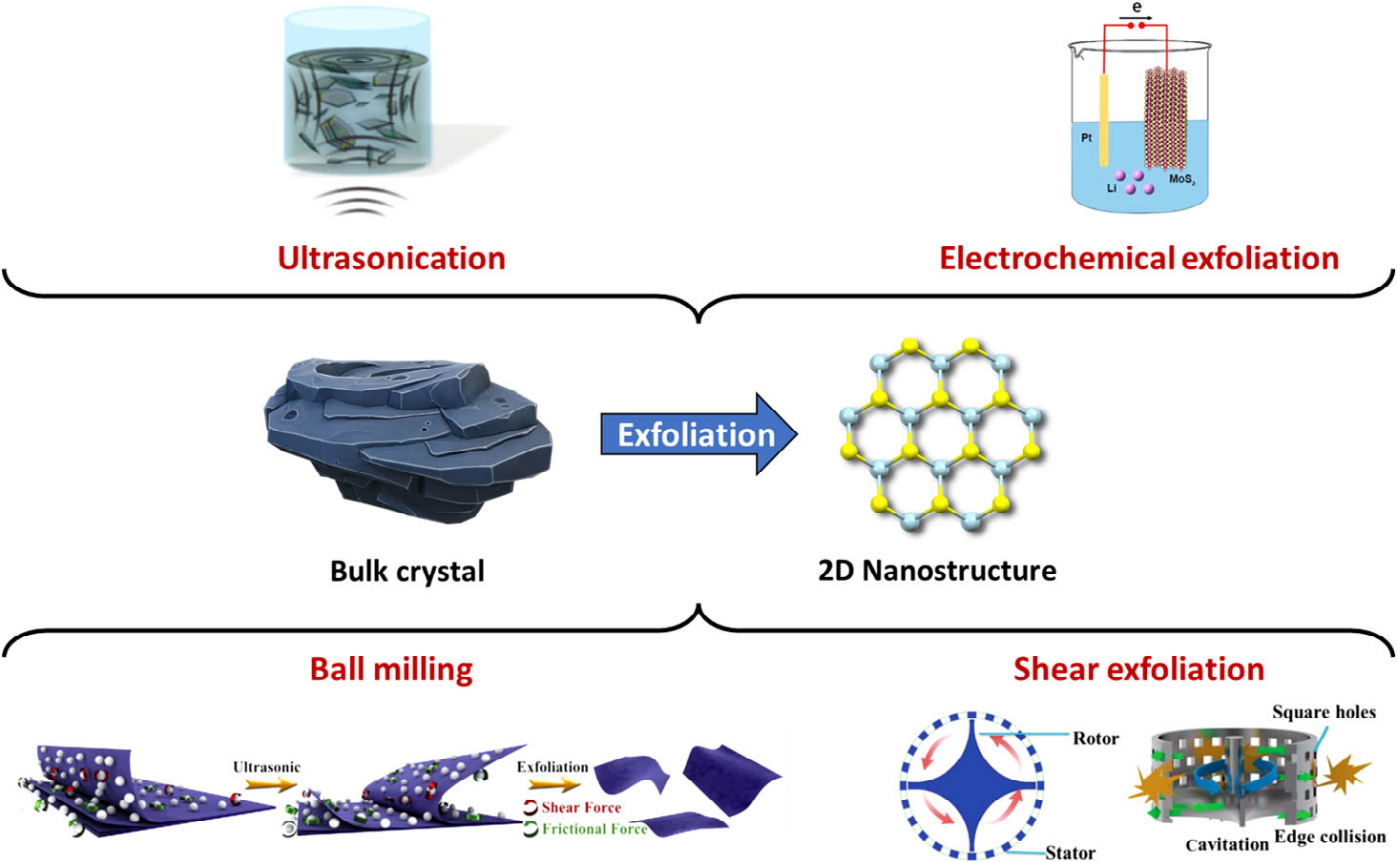
Illustration of various LPE methods (top‐down approaches) to obtain solution‐processed TMDs, including ultrasonication, electrochemical exfoliation, ball milling, and shear exfoliation. Ball milling: Reproduced with permission.^[^ ^29^ ^]^ Copyright 2020, Wiley‐VCH. Shear exfoliation: Reproduced with permission.^[^ ^30^ ^]^ Copyright 2016, Royal Society of Chemistry. Ultrasonication: Reproduced with permission.^[^ ^36^ ^]^ Copyright 2017, American Chemical Society. Electrochemical exfoliation: Reproduced with permission.^[^ ^35^ ^]^ Copyright 2018, Elsevier.

Any successful application of 2DMs is strictly related to the quality and yield of the employed production methods. To date, micromechanical cleavage remains the most straightforward source of high‐quality TMDs, although it suffers from low yields and production rates that are not compatible with (industrial) large‐scale production. In light of such limitations, during the last decade, solution processing (also referred to as liquid‐phase exfoliation (LPE)) has become the prime alternative to obtain high‐concentration and high‐volume TMD inks, thereby offering an efficient and versatile production of 2D structures.^[^ ^25^, ^26^ ^]^ The different possible approaches can be classified as bottom‐up and top‐down: in the former, TMDs are synthesized starting from suitable chemical precursors and by binding atoms to each other, whereas in the latter, TMDs are exfoliated from the respective bulk crystals under specific conditions. The top‐down approaches (**Figure** **1**) guarantee a higher throughput and the best trade‐off among key parameters that can be assessed when evaluating production protocols in 2DM science, such as cost, purity, scalability, yield, and so on.^[^ ^27^ ^]^ These methods entail a typical process flowchart, involving (i) the dispersion of bulk materials in a specific solvent, (ii) the exfoliation of the bulk crystals through (acoustic) cavitation or shear forces, and (iii) the sorting by size of the exfoliated flakes.^[^ ^17^ ^]^ In recent years, various methods have been developed to obtain solution‐processed 2DMs, such as ball milling,^[^ ^28^, ^29^ ^]^ shear exfoliation,^[^ ^30^, ^31^, ^32^ ^]^ wet‐jet milling,^[^ ^33^ ^]^ microfluidization,^[^ ^34^ ^]^ and electrochemical exfoliation.^[^ ^35^ ^]^ Nevertheless, among top‐down strategies, ultrasonication‐assisted exfoliation of bulk TMD crystals is the prototypical LPE method,^[^ ^36^ ^]^ where the starting material is subjected to vibration and cavitation forces (propagating within the solvent) that overcome the weak vdW interactions among adjacent sheets forming layered structures, thereby peeling them away. In addition to mechanistic considerations, several parameters play a crucial role in determining the quality and efficiency of the exfoliation procedures, such as power, time, and type of sonication (e.g., sonic baths or sonic probes), as well as the exfoliation solvent.^[^ ^16^, ^37^ ^]^ It is worth emphasizing that the choice of the solvent is of paramount importance as it plays a triple role: (i) it is the medium designed for the propagation of the ultrasound waves; (ii) it shall exhibit suitable physicochemical parameters to minimize the mixing enthalpy between the liquid and the layered structures, promoting solvent intercalation, thereby weakening the interlayer vdW interactions; and (iii) it stabilizes the exfoliated materials owing to steric barrier effects, preventing reaggregation phenomena.^[^ ^38^ ^]^ By the same token, various LPE approaches have been developed to employ less hazardous and lower boiling point solvents, entailing the use of solvent/surfactant mixtures.^[^ ^26^ ^]^ Although these additivities provide some benefits, modulating the surface tension of the solvent, improving the flake dispersibility, and preventing reaggregation phenomena,^[^ ^39^, ^40^ ^]^ it is extremely challenging to get rid of them from the exfoliated materials, affecting their properties such as the electrical performance.^[^ ^41^ ^]^ The recent advances on deposition techniques of solution‐processed TMDs have further promoted their use in the form of colloidal dispersions, coating, and thin films produced by inkjet‐printing, spray coating, roll‐to‐roll, screen printing, spin‐coating, drop‐casting, etc.^[^ ^17^, ^25^ ^]^ Finally, due to its scalability and cost‐effectiveness, LPE can efficiently provide TMDs in massive quantities, paving the way for their application in several research fields.

## 3 Landscape of Defects in TMDs

**Figure 2:**
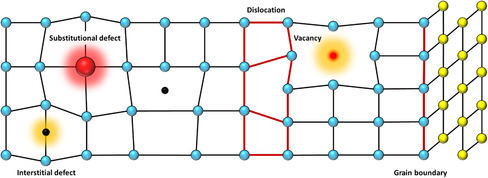
Illustration of the typical defects encountered within the crystal lattices of solution‐processed TMDs.

Crystal structures are built up by repeated translation of the basic unit cell along the three crystallographic axes. However, as a result of the interplay between thermodynamics and kinetics of the materials processing, a crystal with a perfectly regular arrangement of atoms cannot exist; imperfections, irregularities, and defects are present to some extent in all crystals. The formation and evolution of defects become more critical at the nanoscale, as their interaction with interfaces plays a major role in determining the final physicochemical properties, especially in 2DMs due to their extremely high surface‐to‐volume ratio. The landscape of defects in TMDs is quite broad and complex, as a great variety of phenomena might take place affecting their features (e.g., density, size, stability). In particular, such 2DMs exhibit both topological and structural defects generated during their synthetic or exfoliation process (in the latter case, defects might also be inherited from the bulk crystals). The main defects encountered in TMDs according to their atomic structure and dimensionality (**Figure** **2**) are presented and discussed in the following sections.

### 3.1 0D Defects

A point defect is an irregularity in the crystal lattice related to a dangling bond, a missing atom (vacancy), an extra atom (interstitial), or an impurity atom. At temperature *T* ≠ 0 K, there is always a thermodynamically stable concentration of vacancies and interstitial atoms. The number of defects at the equilibrium for a certain *T* can be determined as follows:

(1) $$N_{d}=N\exp(\frac{-E_{d}}{k_{B}T})\;$$

where *N*_d_ is the number of defects, *N* is the total number of atomic sites, *E*_d_ is the activation energy necessary to form the defect, *k*_B_ is the Boltzmann constant, and *T* is the absolute temperature. Hence, vacancy concentration increases with the temperature; in fact, as thermal energy increases, each atom's probability of jumping out from its lowest‐energy position also increases. Atoms occupying positions within the lattice that are generally unoccupied in perfect crystals are called interstitial defects, whose formation energy is considerably higher than a vacancy's one. Therefore, the equilibrium density of interstitials is several orders of magnitude lower than that of vacancies. Moreover, if the interstitial atom is much larger than the rest of the atoms in the crystal, it will push the surrounding atoms further apart and distort the lattice planes. Interstitial atoms in TMDs might be produced by severe local distortion during plastic deformation or by irradiation with high‐energy particles, strongly affecting the properties of the resulting materials.^[^ ^42^, ^43^, ^44^ ^]^ In addition, when an atom occupies a nearby interstitial position, leaving a vacancy at the original lattice site, this is known as Frenkel defect.

**Figure 3:**
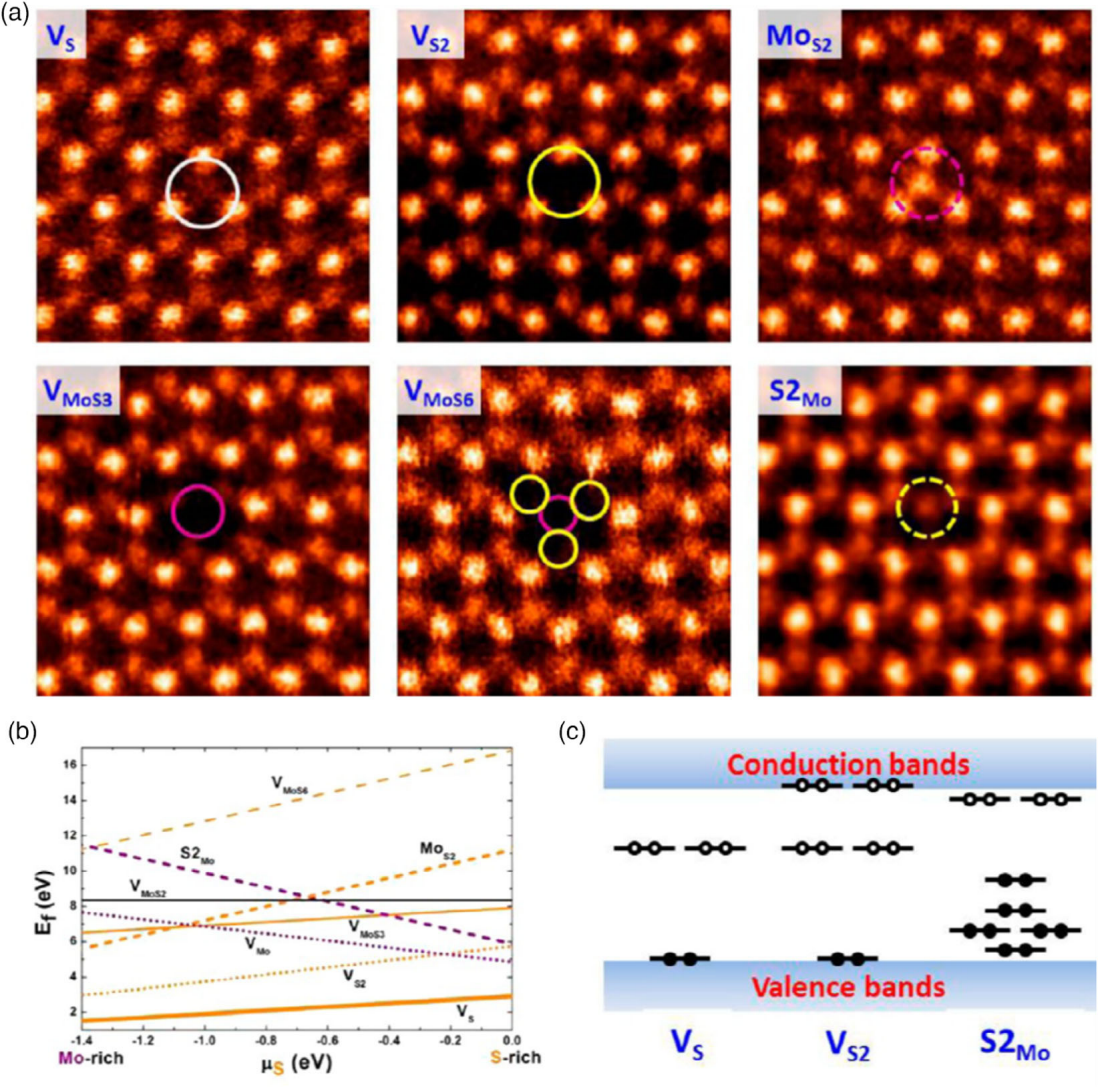
a) Atomic resolution images of different intrinsic point defects present in monolayer MoS_2_ grown by chemical vapor deposition. b) Formation energies of different point defects as a function of sulfur chemical potential. c) Schematic representation of defect levels within the MoS_2_ band structure. Reproduced with permission.^[^ ^45^ ^]^ Copyright 2013, American Chemical Society.

Taking into account the thermodynamic parameters (e.g., formation energy) related to each of the abovementioned crystallographic 0D defects, chalcogen vacancies (e.g., sulfur vacancies, *V*_S_) represent the simplest and most abundant structural irregularities in solution‐processed TMDs. Considering MoS_2_ as a prototypical case, six different varieties might be observed (**Figure** **3**): single *V*_S_, double *V*_S_ (*V*_S2_), vacancy of an Mo atom and a triad of its bonded S atoms within one plane (*V*_MoS3_), vacancy of an Mo atom and all six of its nearest neighbors (*V*_MoS6_), an antisite with an Mo atom occupying a double *V*_S_ (Mo_S2_), and a pair of S atoms occupying an Mo position (S2_Mo_).^[^ ^45^ ^]^ Among them, single *V*_S_ has the lowest formation energy (≈2 eV), although the exact value depends on the chemical potential of Mo and S atoms under different conditions.^[^ ^46^ ^]^

### 3.2 1D Defects

The principal 1D crystal defects encountered in TMDs are dislocations. In a hypothetical perfect crystal, the atoms lie in planes within the lattice; however, if half‐plane of atoms is missing, a line defect exists along the bottom edge of the half‐plane that remains.^[^ ^47^ ^]^ Line defects are also known as dislocations because atoms are displaced from their positions within crystal lattices. Two types of dislocations exist: the pure edge and the pure screw dislocations. Edge dislocations consist of an extra half‐plane of atoms within the crystal structure, whereas screw dislocations occur when one edge of the crystal undergoes shear stress and moves, at least, one interplanar distance, while the other edge does not move from its initial position. In general, dislocations consist of a combination of edge and screw, and they are either present as loops or they terminate at the grain boundaries or the free crystal surface.^[^ ^42^ ^]^ Furthermore, in MoS_2_, for instance, as S—S and Mo—Mo homo‐bonds are energetically less favorable than S—Mo hetero‐bonds, dislocations are able to react with *V*_S_ or interstitial S to form dislocation‐vacancy or dislocation‐interstitial complexes.^[^ ^48^ ^]^

**Figure 4:**
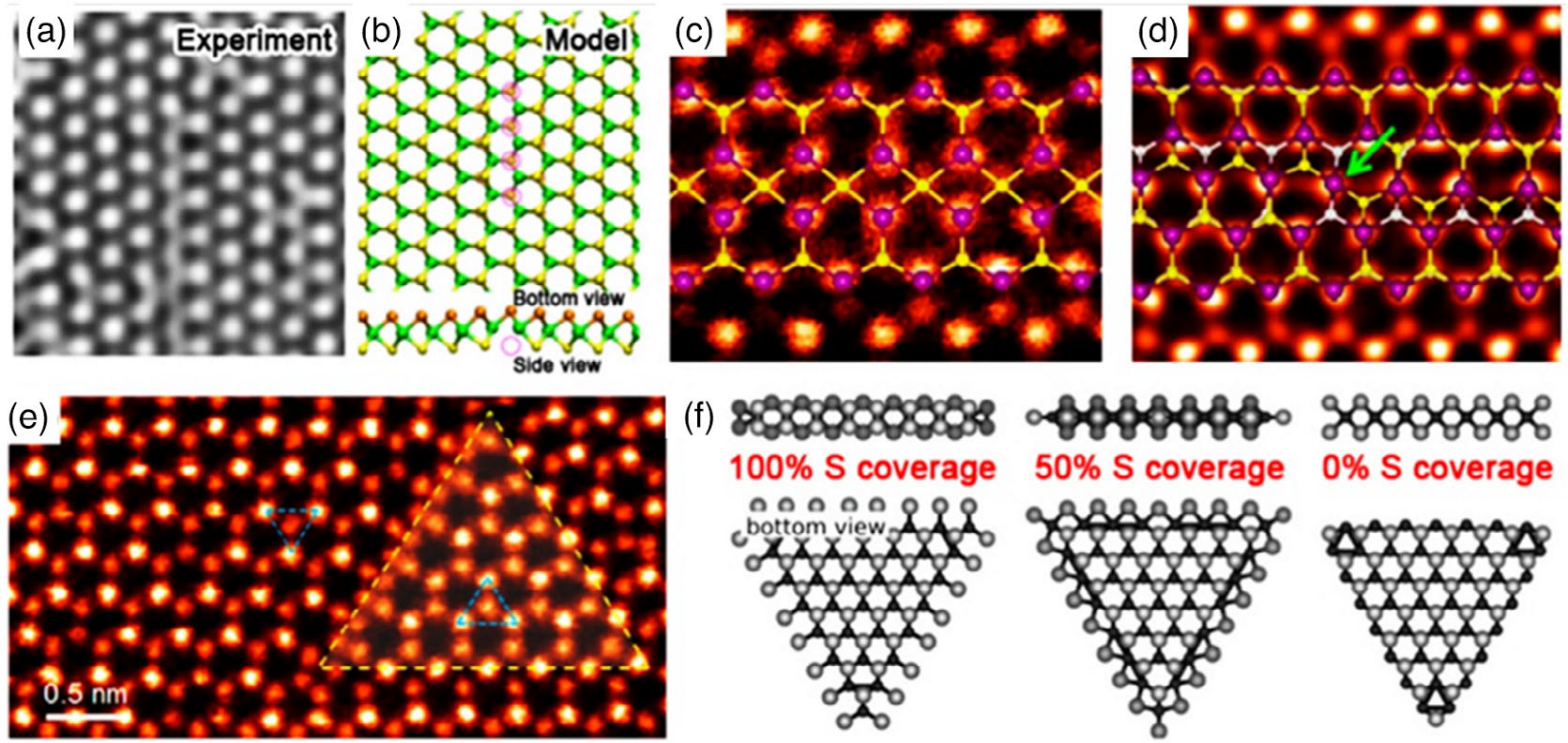
a) High‐resolution transmission electron microscopy (HR‐TEM) image and b) structural model showing a single vacancy line in mechanically exfoliated MoS_2_ monolayer. c,d) Annular dark‐field (ADF) images of grain boundaries in MoS_2_ monolayer grown by chemical vapor deposition. e) ADF image showing an inversion domain in mechanically exfoliated MoSe_2_ monolayer. f) Structural models showing Mo‐terminated MoS_2_ domain edges with different percentages of sulfur coverage. Reproduced under the terms of the CC‐BY 3.0 license.^[^ ^21^ ^]^ Copyright 2016, The Authors, published by IOP Publishing.

Various 1D defects can be found in TMDs, and their rigorous characterization and study rely on advanced electron microscopy techniques (**Figure** **4**). For instance, extrinsic sulfur line vacancies result from the aggregation of *V*_S_ (Figure 4a,b), usually produced by electron bombardment,^[^ ^49^ ^]^ whose formation energy depends on the number of vacancies involved in the process, ranging from 5 to 6 eV per vacancy for an overall length of 6–16 *V*_S_.^[^ ^50^ ^]^ Moreover, strain might be used to induce specific line vacancy orientations in TMDs, tuning the final features such as electronic properties.^[^ ^49^ ^]^ In addition to sulfur line vacancies and dislocations, grain boundaries (Figure 4c,d) are additional and abundant 1D defects encountered in TMDs. When atoms are removed from the crystal lattice, the structure relaxes in three dimensions to form dislocations with different motifs strongly dependent on the angle of grain boundaries.^[^ ^47^, ^51^ ^]^ Finally, the prime 1D defects in TMD nanosheets are represented by their edges, whose energy and composition are related to operating conditions employed during the production steps. For instance, nanoscale calculations predict that under sulfur‐rich conditions, Mo edges with either 50% or 100% S are the most thermodynamically stable, although additional compositions (0% and 50% S) were also observed (Figure 4f).^[^ ^52^, ^53^ ^]^

### 3.3 2D Defects

**Figure 5:**
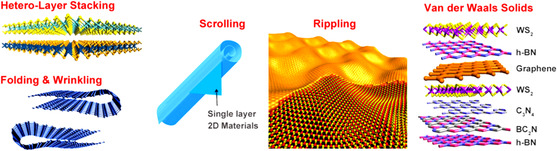
Schematic illustration of the typical 2D defects encountered in TMDs, such as folding, wrinkling, scrolling, rippling, and vertically stacked vdW solids. Reproduced under the terms of the CC‐BY 3.0 license.^[^ ^21^ ^]^ Copyright 2016, The Authors, published by IOP Publishing.

The surface of 2D crystals is prone to form corrugations or, in the worst case, break (e.g., cracks, holes) because of thermal fluctuations involving edge instabilities, strain, thermal vibrations, and contractions. As a result, wrinkles and ripples might be formed, influencing the overall electronic structure and altering the surface properties (**Figure** **5**).^[^ ^54^, ^55^ ^]^ For the sake of clarity, the classification of such corrugations can be simplified by considering their aspect ratio, topology, and order. Wrinkles present a width between 1 and 10s nm, height below 15 nm, and length above 100 nm (aspect ratio > 10); ripples are more isotropic and show feature size below 10 nm (aspect ratio ≈ 1). Moreover, a dense formation and packing of ripples and wrinkles in two or three dimensions generates crumples. Such 2D defects can also be the result of post‐synthetic procedures, when, for instance, TMD nanosheets are stacked manually via transfer techniques and might fold themselves up, heavily affecting their electronic and optical properties.^[^ ^56^, ^57^ ^]^ Finally, by considering the analogies about structural features and interlayer interactions, TMD flakes can be stacked one on top of another forming vertical homo‐ or heterostructures, also known as vdW solids.^[^ ^58^, ^59^ ^]^ Such interfaces can be considered as 2D defects, and the overall material properties are strictly related to those of each component (e.g., lattice mismatch leading to Moiré patterns).^[^ ^60^, ^61^ ^]^

## 4 Generation and Control of Defects in Solution‐Processed TMDs

As already discussed in the previous section, defects are ubiquitous in all materials, and their crucial influence on the physicochemical properties cannot be neglected. The presence of a great variety of defects in TMDs has recently motivated the scientific community to develop new strategies to control their nature and density, tuning the properties of 2DMs on demand according to the envisioned applications. To date, we have a long list of different approaches for solution‐processed TMDs, by exploiting both in situ and ex situ defect engineering strategies. The former rely on synthetic steps carefully designed to accomplish the growth of materials under precise conditions, targeting specific stoichiometric, structural, and crystalline features; conversely, the latter strategies exploit post‐growth treatments to attain the desired defect density and nature, as well as material properties. A further brief description of the most common methods to generate and control defects in solution‐processed TMDs is reported in the following sections.

### 4.1 In Situ Generation of Defects

LPE represents a versatile and efficient option to control the defect density and nature of TMDs. For example, the wafer‐scale in situ growth of vacancy‐tunable TMD films was reported by Lee et al., highlighting the crucial role played by the precursor structure and molar ratio to produce *V*_S_‐modulated 2D crystals.^[^ ^62^ ^]^ In this regard, the presence of chalcogen vacancies induces an upshift of the Fermi level, enhancing the electrochemical catalytic activity in hydrogen evolution reactions (HERs) due to a smaller energy difference with the standard reduction potential of H_2_O/H_2_. Similarly, Xie et al. reported a solvothermal synthetic approach to produce defect‐rich MoS_2_ nanosheets, pointing out a scalable pathway to accomplish the defect engineering on TMD surfaces to expose catalytically active edge sites.^[^ ^22^ ^]^ The synthesis entails the use of a high amount of precursor, hexaammonium heptamolybdate tetrahydrate (NH_4_)_6_Mo_7_O_24_•4H_2_O, and different amounts of thiourea to achieve a controlled modulation of defects in MoS_2_ nanosheets. In particular, thiourea plays a double role acting as (i) reducing agent to form Mo(IV) as well as (ii) efficient additive to stabilize the colloidal dispersion upon adsorption on the surface of primary nanocrystallites, hindering the oriented crystal growth and, therefore, leading to the formation of defect‐rich structures. Finally, Tang et al. reported the synthesis of defect‐rich MoS_2_‐based composite materials for HERs in an alkaline medium.^[^ ^63^ ^]^ In this regard, the use of polyoxometalates as templates and anchor sites for metal salt precursors is beneficial to expose abundant edges with enhanced catalytic activity. Moreover, the addition of impurities via Co‐doping within the MoS_2_ crystal lattice promotes and facilitates the electron transfer from Co to Mo atoms, thereby accelerating the overall HER catalytic process.

### 4.2 Ex Situ Generation of Defects

Owing to the challenging control of defect formation during the synthetic steps, post‐growth (ex situ) treatments have been devised to further develop defect engineering strategies. However, upon production, solution‐processed TMDs already exhibit high defect density due to the inherent features of the related bottom‐up and top‐down LPE approaches, where harsh operating conditions and/or energetic phenomena are involved (e.g., vibration and cavitation forces), promoting the generation of defective structures.^[^ ^20^ ^]^ For this reason, thus far, most of the ex situ methods to generate defects have been employed for TMDs obtained via chemical vapor deposition and micromechanical cleavage, although they represent a viable and appealing option for solution‐processed materials as well.^[^ ^64^, ^65^ ^]^ Thermal annealing has been proved to be a simple strategy to create chalcogen atomic defects (e.g., S, Te, Se) in 2D crystals.^[^ ^66^, ^67^ ^]^ Moreover, the density of line defects can be increased either by increasing the annealing temperature or by depositing chalcogen atoms on the defective samples followed by thermal annealing, highlighting the key role of temperature during the defect formation.^[^ ^68^ ^]^ Another approach to produce chalcogen vacancies exploits laser irradiation,^[^ ^69^ ^]^ where the ability to perform micropatterning represents one of the main advantages over thermal annealing.^[^ ^70^ ^]^ Lasers can also be used for thinning TMDs, reaching single‐layer 2D crystals with feature sizes down to 200 nm and arbitrary shapes and patterns,^[^ ^71^ ^]^ paving the way for the fabrication of single‐ and few‐layer nanosheets with different geometries for (opto)electronic applications. A different strategy takes advantage of plasma treatments (e.g., oxygen, argon, SF_6_, CF_4_, CHF_3_)^[^ ^72^ ^]^ to generate a great variety of defects, such as chalcogen vacancies,^[^ ^73^ ^]^ oxygen‐transition metal bonds^[^ ^74^ ^]^ (promoting further chemical functionalization), and ripples affecting the overall electrical device performance.^[^ ^72^ ^]^ Finally, an additional ex situ method to engineer defects in TMDs entails ion bombardment, where several species might be adopted, such as He, Ga, or Ar ions,^[^ ^74^, ^75^, ^76^ ^]^ α‐particles,^[^ ^77^ ^]^ and proton beams.^[^ ^78^ ^]^

## 5 Molecular Chemistry Functionalization Approaches

**Figure 6:**
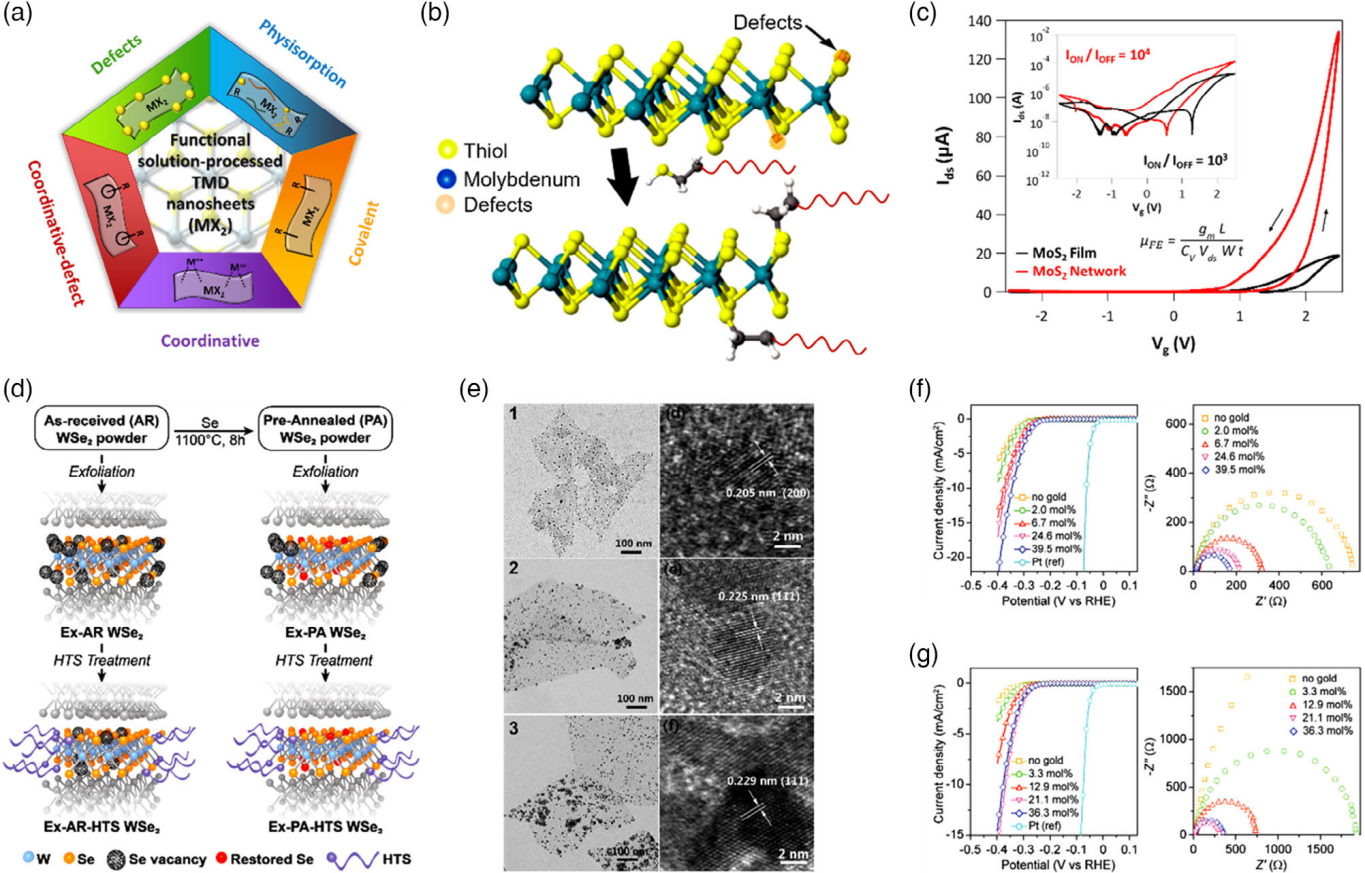
a) Functionalization strategies via molecular chemistry approaches for solution‐processed TMDs. Reproduced with permission.^[^ ^20^ ^]^ Copyright 2019, Royal Society of Chemistry. b) Structural model illustrating the healing of *V*_S_ in MoS_2_ nanosheets using thiolated molecules. Reproduced with permission.^[^ ^79^ ^]^ Copyright 2013, American Chemical Society. c) Typical transfer curves for liquid‐gated thin‐film transistors based on MoS_2_ films and networks, the latter obtained upon exposure of films to dithiolated molecules of 1,4‐benzenedithiol. Inset shows the log‐scale electrical characteristics and the equation used for the calculation of field‐effect mobility. Reproduced with permission.^[^ ^84^ ^]^ Copyright 2021, Springer Nature. d) Illustration of different treatments performed on WSe_2_ nanosheets. A preliminary annealing in the presence of Se powder leads to exfoliated WSe_2_ flakes with lower defect density (Se vacancies). The following treatment with hexyltrichlorosilane is carried out to passivate the edge Se vacancies and improve the photocurrent for solar H_2_ production. Reproduced with permission.^[^ ^64^ ^]^ Copyright 2018, American Chemical Society. e) Transmission electron microscopy images (left) and HR‐TEM images (right) of modified MoS_2_ nanosheets with Ag (1), Pd (2), and Pt (3) nanocrystals. Reproduced with permission.^[^ ^93^ ^]^ Copyright 2014, Royal Society of Chemistry. f) Polarization curves for HER (left) and impedance spectra (right) for MoS_2_ and g) WS_2_ nanosheets with different loading levels of AuNPs. Reproduced with permission.^[^ ^92^ ^]^ Copyright 2013, American Chemical Society.

The previous sections are instructive on the nature and generation of defects in TMDs, as well as inherent features of their production methods and characteristic thermodynamic and kinetic aspects. During the last decade, 2DM scientists have developed innovative molecular chemistry approaches to engineer defects in solution‐processed TMDs, tuning on demand their physicochemical properties according to the envisioned applications. Modern functionalization strategies exploit various mechanisms and interactions, offering a plethora of possibilities in current 2DM science (**Figure** **6**a).

One of the main approaches of defect engineering for solution‐processed TMDs exploits a coordinative‐defect method, capitalizing on the unavoidable presence of chalcogen atom vacancies mainly produced during the exfoliation steps. In this regard, the seminal work was published by Dravid and coworkers in 2013,^[^ ^79^ ^]^ where chemically exfoliated MoS_2_ nanosheets were subjected to reactions with different thiol‐terminated polyethylene glycol derivatives bearing ionic and nonionic headgroups (Figure 6b). The high reactivity between thiol groups and sulfur vacancies leads to the healing process of 2D crystals, where the sulfur atoms of the molecular systems become an integral part of the inorganic lattice.^[^ ^80^ ^]^ The results of the ligand affinity tests have shown that the thiol group was responsible for the observed MoS_2_ modifications: changing the ligand structure (e.g., polarity of the headgroup, conjugation), different colloidal stability, catalytic activity, and chemical affinity toward specific molecules could be achieved. To date, the functionalization of TMD nanosheets via coordinative‐defect approach can be performed by three distinct strategies: (i) the direct functionalization of TMD colloidal dispersions,^[^ ^80^, ^81^ ^]^ (ii) the functionalization of substrate‐supported solution‐processed TMD nanosheets,^[^ ^35^ ^]^ and (iii) the simultaneous exfoliation of bulk systems and functionalization of related exfoliated materials.^[^ ^82^ ^]^ Transition metal disulfide (MS_2_) have drawn great attention because of their versatility and aptitude to undergo healing reactions, in which thiolated molecules are exploited to fill *V*_S_ contained within the crystal structure. In particular, MS_2_ have been studied and processed with dithiolane derivatives and thiolated molecules, to produce hybrid systems characterized by new and/or enhanced properties and performance, spanning from biosensing^[^ ^83^ ^]^ to electronics.^[^ ^35^ ^]^ In this framework, our group has recently reported an innovative functionalization strategy exploiting π‐conjugated dithiolated molecules,^[^ ^84^ ^]^ to simultaneously heal *V*_S_ and bridge adjacent nanosheets in TMD thin films, thereby enhancing the inter‐flake charge transport and the overall electrical performance due to reduced inter‐flake resistance (Figure 6c).^[^ ^85^ ^]^ However, it is worth mentioning that the sulfur healing reaction is not always the most favorable process, and its mechanism is still under debate. In fact, two thiolated molecules can also interact through a TMD‐mediated process and dimerize to form disulfide species that will be physisorbed onto the 2D crystal surface via weak vdW interactions.^[^ ^86^, ^87^ ^]^ A similar approach exploits the high reactivity of chalcogen vacancies in TMDs although their crystal structure is not restored (i.e., no healing reaction). In particular, such a defect passivation method aims to deactivate the defect states without a permanent change in the intrinsic crystal lattice (Figure 6d). Toward this end, the adsorbed molecules should be chemically and thermally stable on TMD surfaces, avoiding any desorption and decomposition process during the fabrication steps. To date, the overwhelming majority of works reported in the literature about defect passivation rely on the formation of an organic–inorganic vdW interface, where a great variety of molecular systems can be exploited to influence and tailor the ultimate properties of TMDs. For instance, Yu et al. investigated the role of defects on the performance of WSe_2_‐based photoelectrodes, highlighting the effects of their passivation with silane molecules.^[^ ^64^ ^]^ In particular, the high reactivity of Se vacancies (introduced during the exfoliation steps) promotes the interaction with the surfactant species, leading to multiple benefits. In fact, while the passivation of surface dangling bonds can reasonably reduce photogenerated charge recombination, the potential self‐polymerization of silane molecules on TMD surfaces^[^ ^88^ ^]^ can also improve the loading of surfactant, affecting the overall electrostatics by creating a surface dipole that might facilitate the charge extraction.^[^ ^89^ ^]^ In the same vein, Tascón and coworkers reported the use of functional biomolecules as dispersing agents for solution‐processed TMD nanosheets.^[^ ^90^ ^]^ DNA and RNA nucleotides act as highly efficient stabilizers, favoring the preparation of aqueous dispersions at very high concentrations (up to 5–10 mg mL^−1^). Such exceptional colloidal stability relies on the specific interactions of Lewis acid–base type between the biomolecules and defective TMDs, where the acidic *V*_S_ in MS_2_ will interact with the basic nucleobases, improving stability and catalytic activity in the reduction of nitroarenes. Finally, the nucleotide‐stabilized nanosheets also show high biocompatibility toward murine preosteoblasts and human sarcoma osteoblasts, paving the way for future applications in drug delivery and cancer treatments under different operating conditions (e.g., temperature, pH). The defective areas of TMD nanosheets are also prone to undergo functionalization reactions via chemisorption, taking advantage of their inherent high reactivity toward a great variety of species. In this regard, an additional and appealing molecular strategy envisages the growth of noble metal nanoparticles (NPs) in defective regions of TMD crystals, such as Au,^[^ ^91^, ^92^ ^]^ Pd,^[^ ^93^, ^94^ ^]^ Pt,^[^ ^95^ ^]^ and AgNPs^[^ ^96^ ^]^ (Figure 6e). The latter can interact with solution‐processed TMDs via covalent and non‐covalent interactions, leading to hybrid systems characterized by exceptional performance for sensing and (photo)catalysis.^[^ ^96^, ^97^ ^]^ The growth of NPs takes place after the reduction of a noble metal precursor salt induced by either the addition of a reducing agent^[^ ^98^ ^]^ or spontaneous formation.^[^ ^92^ ^]^ In the latter case, Kim et al. succeeded in decorating electrochemical exfoliated MoS_2_ and WS_2_ nanosheets with AuNPs, using HAuCl_4_ in water as precursor.^[^ ^92^ ^]^ The functionalization preferentially occurs on the defect sites, mainly located at the edges of the nanosheets and secondarily in their basal planes, more reactive than bulk crystals. A redox process takes place between [AuCl_4_]^−^ ions and TMD materials, and it is induced by the favorable matching of energy levels involved during the reaction, namely the TMD ionization energy (5.4 and 5.2 eV for MoS_2_ and WS_2_, respectively) lying well above the standard reduction potential of HAuCl_4_/Au (+1.0 V versus SHE). It is worth mentioning that AuNPs play a double role, acting as spacers to inhibit restacking phenomena and improving the charge transport among adjacent nanosheets, thereby leading to superior electrocatalytic performance in HERs (Figure 6f,g). Furthermore, the synthesis and growth of metal NPs can also be promoted by the use of suitable reducing agents. For instance, Huang et al. capitalized on solution‐processed MoS_2_ nanosheets to support the epitaxial growth of Pd, Pt, and Ag nanoclusters under ambient conditions, using different reduction methods according to the chosen metal precursor.^[^ ^99^ ^]^ Remarkably, TMD nanosheets are able to address NPs toward an epitaxial growth, resulting in preferential growth orientations such as (100) and (111). Moreover, in addition to providing plenty of nucleation sites in the defective areas, 2D epitaxial templates also stabilize small NPs and prevent them from aggregation. It is worth mentioning that for MoS_2_/PtNPs hybrid systems, the nanomaterials exhibited much better electrocatalytic activity than commercial Pt catalyst, at the same Pt loading, likely due to the presence of additional (110) and (311) facets that promote the catalytic reactions.^[^ ^100^, ^101^ ^]^ TMD nanosheets might also be subjected to prior functionalization reactions by exploiting organohalide^[^ ^96^ ^]^ or thiol molecules,^[^ ^102^ ^]^ to activate the 2D structure and make it more reactive during the following steps of NP growth.^[^ ^97^ ^]^ By and large, the abovementioned functionalization approaches represent cutting‐edge molecular strategies to produce novel and hybrid functional materials with state‐of‐the‐art performance based on defective solution‐processed TMDs.

## 6 Conclusions and Outlook

Solution‐processed TMDs display unique and diverse physicochemical properties, where their defectiveness represents a key aspect to produce hybrid multifunctional materials and boost the technological progress in 2DM science. Molecular chemistry functionalization strategies might also be exploited to further tailor the properties of such hybrid systems according to the envisioned applications, taking advantage of several mechanisms and interactions. More specifically, the virtually infinite number of functionalizing molecules offers a challenging and promising opportunity to develop superior technologies based on multifunctional and/or multiresponsive devices, where external stimuli (e.g., heat, light, temperature, magnetic field) might trigger changes in the properties of molecular systems interacting with 2D crystals.

**Figure 7:**
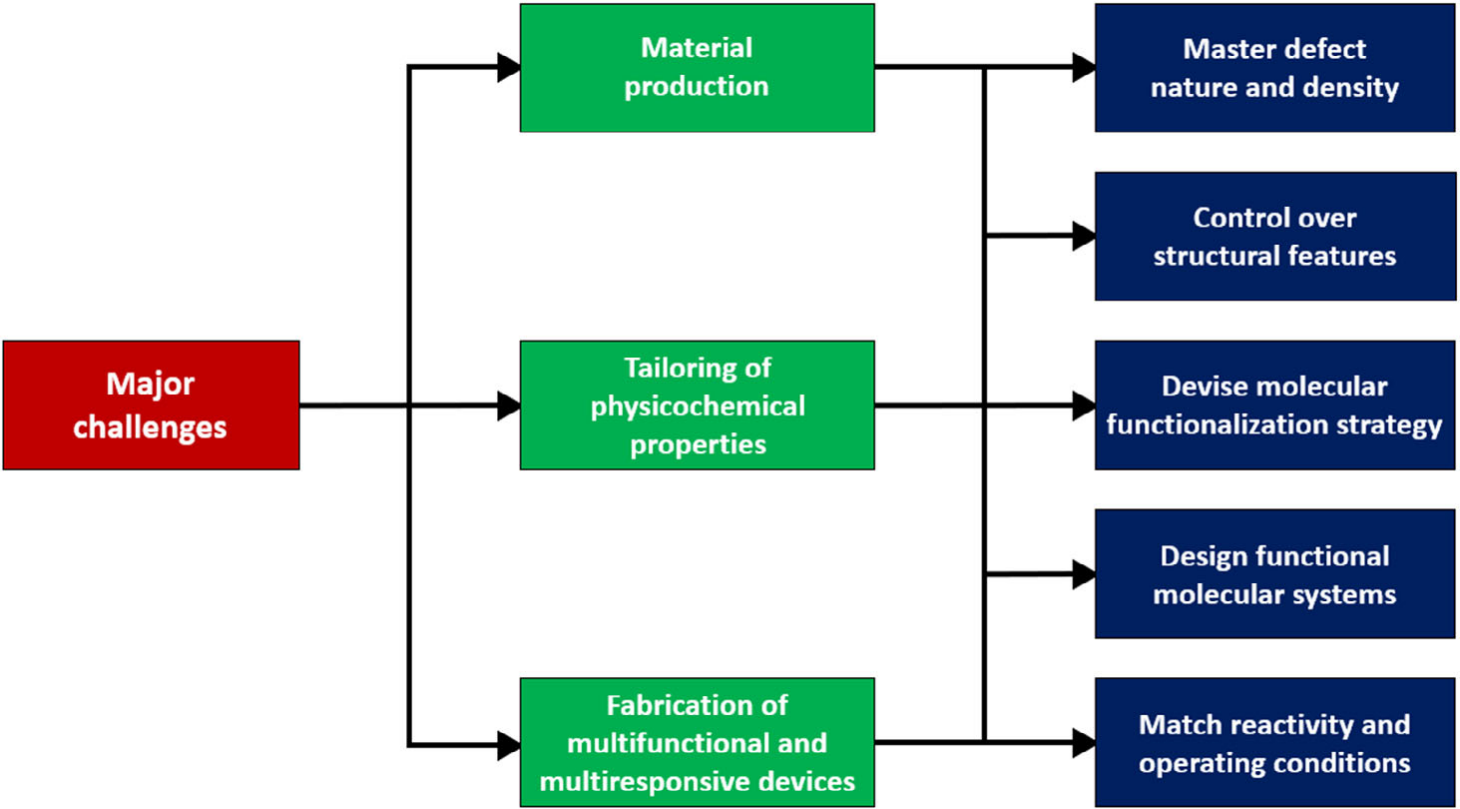
Schematic of the main challenges that need to be tackled in the forthcoming years to promote the progress in 2DM science and enable the development of innovative technologies based on solution‐processed TMDs.

Nevertheless, despite the tremendous and ever‐growing progress of solution‐processed TMDs, a few major challenges still need to be tackled to sustain the production of hybrid systems via defect engineering (**Figure** **7**). (i) A controllable generation of defects, ideally with an atomic precision, including their nature and density, is crucial to assess the structural features of TMD crystals, strongly related to future practical applications. (ii) A reliable characterization and interpretation of defect–property and healing–property relationship is highly desirable, to devise innovative functionalization strategies and molecular systems yielding state‐of‐the‐art device performance. (iii) Pushing the field forward toward broader applications is of paramount importance to endorse the advances of solution‐processed TMDs in currently underrated research areas, such as electronics and optoelectronics, where their performances remain yet moderate.

All in all, the tuning of physicochemical properties via defect engineering represents a smart and elegant alternative to develop original, cutting‐edge, and exotic hybrid technologies based on solution‐processed TMDs. In particular, the forthcoming technological progress shall exploit the huge potential of these emerging materials, flagship systems in 2DM science, with a specific focus on disruptive technologies in flexible and wearable (opto)electronics, (bio)sensing, and (photo)catalysis.

## Conflict of Interest

The authors declare no conflict of interest.
